# Supplementary material for: Risk and predictive factors for severe dengue infection: A systematic review and meta-analysis
Source: PLoS One. 2022 Apr 15;17(4):e0267186. doi: 10.1371/journal.pone.0267186 (PMC9012395; doi:10.1371/journal.pone.0267186)
Supplement: S1 Table — (DOC) [file pone.0267186.s002.doc]

**Table S1 Scoring system for quality assessment of selected studies**

| **Criteria** | **0 point** | **1 point** |
| --- | --- | --- |
| Data collection | Retrospective, or no description | Prospective or cross-section |
| Assignment of the patient | Not consecutive/random, or no description | Consecutive or random |
| Inclusion criteria | No description | Full description |
| Exclusion criteria | No description | Full description |
| Characteristic of patient population | No description | Full description |
| Interpretation of other characteristic | No description | Full description |
| Method quality | No description | Description method for DF and SDD groups |
| Interpretation of factors | Not blinded or no description | Description of blinded method |
| Dengue diagnosis | No description | Full description |
