# Supplementary material for: Risk and predictive factors for severe dengue infection: A systematic review and meta-analysis
Source: PLoS One. 2022 Apr 15;17(4):e0267186. doi: 10.1371/journal.pone.0267186 (PMC9012395; doi:10.1371/journal.pone.0267186)
Supplement: S2 Table — (DOCX) [file pone.0267186.s003.docx]

**Table S2 The scores of studies included in this meta-analysis according to NOS**

| **Study ID** | **First author** | **Data collection** | **Assignment of the patient** | **Inclusion criteria** | **Exclusion criteria** | **Characteristic of patient population** | **Interpretation of other characteristic** | **Method quality** | **Interpretation of factors** | **Dengue diagnosis** | **Quality score** |
| --- | --- | --- | --- | --- | --- | --- | --- | --- | --- | --- | --- |
| 1 | Federico P C | 0 | 1 | 1 | 0 | 0 | 0 | 1 | 1 | 1 | 5 |
| 2 | Vipa Thanachartwet | 1 | 1 | 1 | 1 | 1 | 1 | 1 | 1 | 1 | 9 |
| 3 | Nguyen Minh Tuan | 1 | 1 | 1 | 1 | 1 | 1 | 1 | 1 | 1 | 9 |
| 4 | Yong Ping Lin | 0 | 1 | 1 | 0 | 1 | 0 | 1 | 1 | 1 | 6 |
| 5 | Samitha Fernando | 0 | 1 | 1 | 1 | 1 | 1 | 1 | 1 | 1 | 8 |
| 6 | Vipa Thanachartwet | 1 | 1 | 1 | 1 | 1 | 0 | 1 | 1 | 1 | 8 |
| 7 | Baolin Liao | 0 | 1 | 0 | 0 | 1 | 0 | 1 | 1 | 1 | 5 |
| 8 | Amita Jain | 1 | 1 | 0 | 0 | 1 | 0 | 1 | 1 | 1 | 6 |
| 9 | Kumar S Abhishek | 1 | 1 | 0 | 0 | 0 | 0 | 1 | 0 | 1 | 4 |
| 10 | Diana Giraldo | 0 | 1 | 1 | 0 | 0 | 0 | 1 | 1 | 1 | 5 |
| 11 | Laura B. Talarico | 0 | 1 | 1 | 0 | 1 | 0 | 1 | 1 | 1 | 6 |
| 12 | Chien-Chih Chen | 0 | 1 | 1 | 1 | 0 | 0 | 0 | 1 | 1 | 5 |
| 13 | Mohit Singla | 1 | 1 | 1 | 0 | 1 | 0 | 1 | 1 | 1 | 7 |
| 14 | Elzinandes de Azeredo | 0 | 1 | 1 | 0 | 1 | 1 | 1 | 1 | 1 | 7 |
| 15 | Anyelo Dura´n | 0 | 0 | 1 | 0 | 1 | 1 | 1 | 1 | 1 | 6 |
| 16 | K. S. Sahana | 1 | 1 | 1 | 0 | 0 | 0 | 1 | 1 | 0 | 5 |
| 17 | Julia Arias | 0 | 0 | 1 | 0 | 1 | 0 | 1 | 1 | 1 | 5 |
| 18 | B. Hoffmeister | 0 | 1 | 1 | 1 | 0 | 0 | 1 | 1 | 1 | 6 |
| 19 | Renato Antônio Oliveira | 0 | 1 | 1 | 0 | 1 | 0 | 1 | 1 | 1 | 6 |
| 20 | Sergio Isaac Cruz Hernández | 0 | 1 | 1 | 0 | 0 | 0 | 1 | 1 | 1 | 5 |
| 21 | Sing-Sin Sam | 0 | 1 | 1 | 0 | 0 | 0 | 1 | 1 | 1 | 5 |
| 22 | Hope H. Biswas | 1 | 1 | 1 | 0 | 1 | 0 | 1 | 1 | 1 | 7 |
| 23 | sergio Isaac De La Cruz H | 0 | 0 | 0 | 0 | 0 | 0 | 1 | 1 | 1 | 3 |
| 24 | Yadunanda Kumar | 1 | 1 | 1 | 0 | 0 | 0 | 1 | 1 | 1 | 6 |
| 25 | Jien-Wei Liu | 0 | 1 | 1 | 1 | 1 | 0 | 1 | 1 | 1 | 7 |
| 26 | Gabriela M. Marón | 1 | 1 | 1 | 0 | 1 | 0 | 1 | 1 | 1 | 7 |
| 27 | D. Priyadarshini | 0 | 1 | 1 | 0 | 1 | 1 | 1 | 1 | 1 | 7 |
| 28 | Erum Khan | 0 | 1 | 1 | 0 | 1 | 0 | 1 | 1 | 1 | 6 |
| 29 | Annette Fox | 1 | 1 | 1 | 0 | 1 | 0 | 1 | 1 | 1 | 7 |
| 30 | Jessica R. Fried | 1 | 1 | 1 | 1 | 1 | 0 | 1 | 1 | 1 | 8 |
| 31 | Luis Angel Villar-Centeno | 1 | 1 | 1 | 1 | 0 | 0 | 1 | 1 | 1 | 7 |
| 32 | Nasim A. Khana | 0 | 1 | 1 | 0 | 0 | 0 | 1 | 1 | 1 | 5 |
| 33 | Celia C. Carlos | 0 | 1 | 1 | 0 | 0 | 0 | 1 | 1 | 1 | 5 |
| 34 | Lien-Cheng Chen | 0 | 1 | 1 | 0 | 1 | 0 | 1 | 1 | 1 | 6 |
| 35 | Adriana O. Guilarde | 1 | 1 | 1 | 0 | 1 | 0 | 1 | 1 | 1 | 7 |
| 36 | Fernando A Bozza | 0 | 1 | 1 | 0 | 1 | 0 | 1 | 1 | 1 | 6 |
| 37 | S. Anatapreecha | 0 | 1 | 1 | 0 | 0 | 0 | 1 | 1 | 1 | 5 |
| 38 | Wei-Kung Wang | 0 | 1 | 1 | 0 | 0 | 0 | 1 | 1 | 1 | 5 |
| 39 | Eva Harris | 1 | 1 | 1 | 0 | 1 | 0 | 1 | 1 | 1 | 7 |
| 40 | David W. Vaughn | 1 | 1 | 1 | 0 | 0 | 0 | 1 | 1 | 1 | 6 |
| 41 | M. Juffrie, G. M | 0 | 1 | 1 | 0 | 1 | 0 | 1 | 1 | 1 | 6 |
| 42 | E. Villamor | 1 | 1 | 1 | 1 | 0 | 0 | 1 | 1 | 1 | 7 |
| 43 | Lucy Chai See Lum | 0 | 1 | 1 | 0 | 0 | 0 | 0 | 1 | 0 | 3 |
| 44 | Chatporn Kittitraku | 0 | 1 | 1 | 0 | 1 | 0 | 1 | 1 | 0 | 5 |
| 45 | Bernadette Murgue | 0 | 1 | 0 | 0 | 0 | 0 | 1 | 1 | 0 | 3 |
| 46 | Wipawee Jampangern | 0 | 1 | 0 | 0 | 1 | 0 | 1 | 1 | 1 | 5 |
| 47 | Agus Suwandono | 0 | 1 | 1 | 0 | 1 | 0 | 1 | 1 | 1 | 6 |
| 48 | N. Voraphani | 0 | 1 | 1 | 1 | 1 | 0 | 1 | 1 | 0 | 6 |
| 49 | Alegria Levy | 0 | 1 | 1 | 0 | 1 | 0 | 1 | 1 | 1 | 6 |
| 50 | Muhammad Mehmood Riaz | 1 | 1 | 1 | 0 | 1 | 0 | 1 | 1 | 0 | 6 |
| 51 | Vernon J. Lee | 0 | 1 | 1 | 1 | 1 | 0 | 1 | 1 | 1 | 7 |
| 52 | Farhad F Vasanwala | 1 | 1 | 1 | 1 | 0 | 0 | 1 | 1 | 0 | 6 |
| 53 | Junxiong Pang | 0 | 1 | 0 | 0 | 1 | 0 | 1 | 1 | 1 | 5 |
| 54 | Junxiong Pang | 0 | 1 | 1 | 0 | 1 | 1 | 1 | 1 | 1 | 7 |
| 55 | Chien-Sen Tseng | 0 | 1 | 1 | 0 | 1 | 0 | 1 | 1 | 1 | 6 |
| 56 | K. Jayashree | 0 | 1 | 0 | 0 | 0 | 0 | 1 | 1 | 0 | 3 |
| 57 | arrayRenato Antonio dos Santos Oliveira | 0 | 1 | 1 | 0 | 1 | 1 | 1 | 1 | 1 | 7 |
| 58 | Andrea L. Conroy | 1 | 1 | 1 | 0 | 0 | 0 | 1 | 1 | 1 | 6 |
| 59 | Min-Sheng Lee | 0 | 1 | 1 | 0 | 1 | 0 | 1 | 1 | 1 | 6 |
| 60 | Muhammad Imran Hasan Khan | 1 | 1 | 1 | 0 | 1 | 0 | 1 | 1 | 1 | 7 |
| 61 | Muhammad Arif Nadeem Saqib | 0 | 1 | 1 | 0 | 0 | 0 | 1 | 1 | 0 | 4 |
| 62 | R. Soundravally | 1 | 1 | 0 | 0 | 1 | 0 | 1 | 1 | 0 | 5 |
| 63 | Cornelia A.M. vandeWeg | 1 | 1 | 1 | 0 | 0 | 0 | 1 | 1 | 1 | 6 |
| 64 | Malik Asif Humayoun | 0 | 1 | 1 | 0 | 1 | 0 | 1 | 1 | 1 | 6 |
| 65 | Tran Nguyen Bich Chau | 1 | 1 | 1 | 0 | 1 | 0 | 1 | 1 | 1 | 7 |
| 66 | Penelopie Koraka | 0 | 1 | 1 | 0 | 1 | 0 | 1 | 1 | 0 | 5 |
| 67 | Ole Wichmann | 0 | 1 | 1 | 0 | 1 | 0 | 1 | 1 | 0 | 5 |
| 68 | Sotianingsih Haryanto | 1 | 1 | 1 | 0 | 0 | 0 | 1 | 1 | 1 | 6 |
| 69 | Goutam Patra | 1 | 1 | 0 | 0 | 0 | 0 | 0 | 1 | 1 | 4 |
| 70 | Tauqeer Hussain Mallhi | 0 | 1 | 1 | 1 | 1 | 1 | 1 | 1 | 1 | 8 |
| 71 | Shiran Ajith Paranavitane | 1 | 1 | 0 | 0 | 0 | 0 | 1 | 1 | 0 | 4 |
| 72 | Thamarasi Senaratne | 1 | 1 | 1 | 0 | 1 | 1 | 0 | 1 | 1 | 7 |
| 73 | Creuza Rachel Vicente | 1 | 1 | 0 | 0 | 1 | 0 | 1 | 1 | 1 | 6 |
| 74 | Sheila Moura Pone | 0 | 1 | 1 | 1 | 1 | 1 | 0 | 1 | 1 | 7 |
| 75 | Wang WH | 1 | 1 | 1 | 1 | 1 | 0 | 1 | 1 | 1 | 8 |
| 76 | Temprasertrudee S | 0 | 1 | 1 | 1 | 1 | 0 | 1 | 1 | 1 | 7 |
| 77 | Kuo HJ | 0 | 1 | 1 | 0 | 1 | 0 | 1 | 1 | 1 | 6 |
| 78 | Patra G | 0 | 1 | 1 | 0 | 1 | 0 | 1 | 1 | 1 | 6 |
| 79 | Agrawal VK | 1 | 1 | 0 | 0 | 1 | 0 | 1 | 1 | 1 | 6 |
| 80 | Sehrawat P | 1 | 1 | 1 | 1 | 0 | 0 | 1 | 1 | 1 | 7 |
| 81 | Patra G | 1 | 1 | 1 | 0 | 1 | 0 | 1 | 1 | 1 | 7 |
| 82 | Jayarajah U | 0 | 1 | 1 | 0 | 1 | 0 | 1 | 1 | 0 | 5 |
| 83 | Adil B | 1 | 1 | 1 | 1 | 0 | 0 | 1 | 1 | 0 | 6 |
| 84 | Hegazi MA | 0 | 1 | 1 | 1 | 1 | 1 | 1 | 1 | 1 | 8 |
| 85 | Shyamali NLA | 0 | 1 | 0 | 1 | 1 | 1 | 1 | 1 | 1 | 7 |
| 86 | Maneerattanasak S | 0 | 1 | 1 | 1 | 1 | 1 | 1 | 1 | 0 | 7 |
| 87 | Ayaz, Fatima | 1 | 1 | 1 | 1 | 0 | 0 | 0 | 1 | 0 | 5 |

Studies included:

1. Perdomo-Celis F., Salgado D.M., Narvaez C.F., et al. 2017. Magnitude of viremia, antigenemia and infection of circulating monocytes in children with mild and severe dengue. Acta tropica. 167, 1-8.

2. Thanachartwet V., Desakorn V., Sahassananda D., et al. 2016. Serum Procalcitonin and Peripheral Venous Lactate for Predicting Dengue Shock and/or Organ Failure: A Prospective Observational Study. PLoS neglected tropical diseases. 10(8), e0004961.

3. Tuan N.M., Nhan H.T., Chau N.V.V., et al. 2017. An Evidence-Based Algorithm for Early Prognosis of Severe Dengue in the Outpatient Setting. Clinical infectious diseases. 64(5), 656-663.

4. Lin Y.P., Luo Y.S., Chen Y., et al.2016. Clinical and epidemiological features of the 2014 large-scale dengue outbreak in Guangzhou city, China. BMC infectious diseases. 16, 102.

5. Fernando S., Wijewickrama A., Gomes L., et al. 2016. Patterns and causes of liver involvement in acute dengue infection. BMC infectious diseases. 16, 319.

6. Thanachartwet V., Oer-Areemitr N., Chamnanchanunt S., et al. 2015. Identification of clinical factors associated with severe dengue among Thai adults: a prospective study. BMC infectious diseases. 15, 420.

7. Liao B., Tang Y., Hu F., et al. 2015. Serum levels of soluble vascular cell adhesion molecules may correlate with the severity of dengue virus-1 infection in adults. Emerging microbes and infections. 4(4), e24.

8. Jain A., Pandey N., Garg R.K., et al. 2013. IL-17 level in patients with Dengue virus infection & its association with severity of illness. Journal of clinical immunology. 33(3), 613-8.

9. Abhishek K.S., Chakravarti A., Baveja C.P., et al. 2017. Association of interleukin-2, -4 and -10 with dengue severity. Indian journal of pathology & microbiology. 60(1), 66-69.

10. Giraldo D., Sant'Anna C., Perisse A.R., et al. 2011. Characteristics of children hospitalized with dengue fever in an outbreak in Rio de Janeiro, Brazil. Transactions of the Royal Society of Tropical Medicine and Hygiene. 105(10), 601-3.

11. Talarico L.B., Byrne A.B., Amarilla S., et al. 2017. Characterization of type I interferon responses in dengue and severe dengue in children in Paraguay. Journal of clinical virology. 97, 10-17.

12. Chen C.C., Lee I.K., Liu J.W., et al. 2015. Utility of C-Reactive Protein Levels for Early Prediction of Dengue Severity in Adults. BioMed Research International. 2015, 1-6.

13. Singla M., Kar M., Sethi T., et al. 2016. Immune Response to Dengue Virus Infection in Pediatric Patients in New Delhi, India--Association of Viremia, Inflammatory Mediators and Monocytes with Disease Severity. PLoS neglected tropical diseases. 10(3), e0004497.

14. De Azeredo E.L., Fiestas Solorzano V.E., De Oliveira D.B., et al. 2017. Increased circulating procoagulant and anticoagulant factors as TF and TFPI according to severity or infecting serotypes in human dengue infection. Microbes and infection. 19(1), 62-68.

15. Duran A., Carrero R., Parra B., et al. 2015. Association of lipid profile alterations with severe forms of dengue in humans. Archives of virology. 160(7), 1687-92.

16. Sahana K.S., Sujatha R. 2015. Clinical profile of dengue among children according to revised WHO classification: analysis of a 2012 outbreak from Southern India. Indian journal of pediatrics. 82(2), 109-13.

17. Arias J., Valero N., Mosquera J., et al. 2014. Increased expression of cytokines, soluble cytokine receptors, soluble apoptosis ligand and apoptosis in dengue. Virology. s452-453, 42-51.

18. Hoffmeister B., Suttorp N., Zoller T. 2015. The revised dengue fever classification in German travelers: clinical manifestations and indicators for severe disease. Infection. 43(1), 21-8.

19. Oliveira R.A., Silva M.M., Calzavara-Silva C.E., et al. 2016. Primary dengue haemorrhagic fever in patients from northeast of Brazil is associated with high levels of interferon-beta during acute phase. Memorias do Instituto Oswaldo Cruz.111(6), 378-84.

20. Cruz Hernandez S.I., Puerta-Guardo H.N., Flores Aguilar H., et al. 2016. Primary dengue virus infections induce differential cytokine production in Mexican patients. Memorias do Instituto Oswaldo Cruz. 111(3), 161-7.

21. Sam S.S., Teoh B.T., Chinna K., et al. 2015. High producing tumor necrosis factor alpha gene alleles in protection against severe manifestations of dengue. International journal of medical sciences. 12(2), 177-86.

22. Biswas H.H., Gordon A., Nunez A., et al. 2015. Lower Low-Density Lipoprotein Cholesterol Levels Are Associated with Severe Dengue Outcome.

PLoS neglected tropical diseases. 9(9), e0003904.

23. De La Cruz Hernandez S.I., Puerta-Guardo H., Flores-Aguilar H., et al.2014. A strong interferon response correlates with a milder dengue clinical condition. Journal of clinical virology. 60(3), 196-199.

24. Kumar Y., Liang C., Bo Z., et al. 2012. Serum proteome and cytokine analysis in a longitudinal cohort of adults with primary dengue infection reveals predictive markers of DHF. PLoS neglected tropical diseases. 6(11), e1887.

25. Liu J.W., Lee I.K., Wang L., et al. 2013. The usefulness of clinical-practice-based laboratory data in facilitating the diagnosis of dengue illness. BioMed Research International. 2013, 1-11.

26. Maron G.M., Clara A.W., Diddle J.W., et al. 2010. Association between nutritional status and severity of dengue infection in children in El Salvador. The American journal of tropical medicine and hygiene. 82(2), 324-9.

27. Priyadarshini D., Gadia R.R., Tripathy A., et al. 2010. Clinical findings and pro-inflammatory cytokines in dengue patients in Western India: a facility-based study. PloS one. 5(1), e8709.

28. Khan E., Kisat M., Khan N., et al. 2010. Demographic and clinical features of dengue fever in Pakistan from 2003-2007: a retrospective cross-sectional study. PloS one. 5(9), e12505.

29. Fox A., Le N., Simmons C., et al. 2011. Immunological and Viral Determinants of Dengue Severity in Hospitalized Adults in Ha Noi, Viet Nam. PLoS neglected tropical diseases. 5(3), e967.

30. Fried J.R., Gibbons R.V., Kalayanarooj S., et al. 2010. Serotype-Specific Differences in the Risk of Dengue Hemorrhagic Fever: An Analysis of Data Collected in Bangkok, Thailand from 1994 to 2006. PLoS neglected tropical diseases. 4(3), e617.

31. Villarcenteno L.A., DíazQuijano, Fredi Alexander, et al. 2008. Biochemical alterations as markers of dengue hemorrhagic fever. The American journal of tropical medicine and hygiene. 78(3), 370-4.

32. Khan N.A., Azhar E.I., El-Fiky S., et al. 2008. Clinical profile and outcome of hospitalized patients during first outbreak of dengue in Makkah, Saudi Arabia. Acta tropica. 105(1), 39-44.

33. Carlos C.C., Oishi K., Cinco M.T., et al. 2005. Comparison of clinical features and hematologic abnormalities between dengue fever and dengue hemorrhagic fever among children in the Philippines. The American journal of tropical medicine and hygiene. 73(2), 435-40.

34. Chen L.C., Lei H.Y., Liu C.C., et al. 2006. Correlation of serum levels of macrophage migration inhibitory factor with disease severity and clinical outcome in dengue patients. The American journal of tropical medicine and hygiene. 74(1), 142-7.

35. Guilarde A.O., Turchi M.D., Siqueira J.B., et al. 2008. Dengue and dengue hemorrhagic fever among adults: clinical outcomes related to viremia, serotypes, and antibody response. The Journal of infectious diseases, 197(6), 817-24.

36. Bozza F.A., Cruz O.G., Zagne S.M., et al. 2008. Multiplex cytokine profile from dengue patients: MIP-1beta and IFN-gamma as predictive factors for severity. BMC Infectious Diseases. 8, 86.

37. Anantapreecha S., Chanama S., A-Nuegoonpipat A., et al. 2005. Serological and virological features of dengue fever and dengue haemorrhagic fever in Thailand from 1999 to 2002. Epidemiology and infection. 133(3), 503-507.

38. Wang W.K., Chen H.L., Yang C.F., et al. 2006. Slower rates of clearance of viral load and virus-containing immune complexes in patients with dengue hemorrhagic fever. Clinical infectious diseases: an official publication of the Infectious Diseases Society of America. 43(8), 1023-30.

39. Harris E., Videa E., Perez L., et al. 2000. Clinical, epidemiologic, and virologic features of dengue in the 1998 epidemic in Nicaragua. The American journal of tropical medicine and hygiene. 63(1-2), 5-11.

40. Vaughn D.W., Green S., Kalayanarooj S., et al. 2000. Dengue viremia titer, antibody response pattern, and virus serotype correlate with disease severity. The Journal of infectious diseases. 181(1), 2-9.

41. Juffrie M., Meer G.M., Hack C.E., et al. 2001. Inflammatory mediators in dengue virus infection in children: interleukin-6 and its relation to C-reactive protein and secretory phospholipase A2. The American journal of tropical medicine and hygiene. 65(1), 70-5.

42. Villamor E., Villar L.A., Lozano A., et al. 2017. Vitamin D serostatus and dengue fever progression to dengue hemorrhagic fever/dengue shock syndrome. Epidemiology and infection. 145(14), 2961-2970.

43. Lum L.C.S., Goh A.Y.T., Chan P.W.K., et al. 2002. Risk factors for hemorrhage in severe dengue infections. The Journal of pediatrics. 140(5), 629-31.

44. Treeprasertsuk S., Kittitrakul C., Silachamroon U., et al. 2003. Liver Function Tests Abnormality and Clinical Severity of Dengue Infection in Adult Patients. American Journal of Gastroenterology, 98(9), S81-S81.

45. Murgue B., Cassar O., Deparis X.. 2001. Plasma concentrations of sVCAM-1 and severity of dengue infections. Journal of medical virology. 65(1), 97-104.

46. Jampangern W., Vongthoung K., Jittmittraphap A., et al. 2007. Characterization of atypical lymphocytes and immunophenotypes of lymphocytes in patients with dengue virus infection. Asian Pacific journal of allergy and immunology. 25(1), 27-36.

47. Suwandono A., Kosasih H., Nurhayati, et al. 2006. Four dengue virus serotypes found circulating during an outbreak of dengue fever and dengue haemorrhagic fever in Jakarta, Indonesia, during 2004. Transactions of the Royal Society of Tropical Medicine and Hygiene.100(9), 855-62.

48. Voraphani N., Theamboonlers A., Khongphatthanayothin A., et al. 2010. Increased level of hepatocyte growth factor in children with dengue virus infection. Annals of tropical paediatrics. 30(3), 213-8.

49. Levy A., Valero N., Espina L.M., et al. 2010. Increment of interleukin 6, tumour necrosis factor alpha, nitric oxide, C-reactive protein and apoptosis in dengue. Transactions of the Royal Society of Tropical Medicine and Hygiene. 104(1), 16-23.

50. Riaz M.M., Muntaz K., Khan M.S., et al. 2009. Outbreak of Dengue Fever in Karachi 2006- a clinical perspective. J Pak Med Assoc. 59(6), 339-344.

51. Lee V.J., Lye D.C.B., Sun Y., et al. 2008. Predictive value of simple clinical and laboratory variables for dengue hemorrhagic fever in adults. Journal of Clinical Virology. 42(1), 34-39.

52. Vasanwala F.F., Puvanendran R., Fook-Chong S., et al. 2011. Could peak proteinuria determine whether patient with dengue fever develop dengue hemorrhagic/dengue shock syndrome? - A prospective cohort study. BMC infectious diseases. 11(1), 212-212.

53. Pang J.X., Salim A., Lee V.J., et al. 2012. Diabetes with Hypertension as Risk Factors for Adult Dengue Hemorrhagic Fever in a Predominantly Dengue Serotype 2 Epidemic: A Case Control Study. PLoS neglected tropical diseases. 6(5), e1641.

54. Pang J.X., Hsu J.P., Yeo T.W., et al. 2017. Diabetes, cardiac disorders and asthma as risk factors for severe organ involvement among adult dengue patients: A matched case-control study. Scientific reports. 7, 39872.

55. Tseng C.S., Lo H.W., Teng H.C., et al. 2005. Elevated levels of plasma VEGF in patients with dengue hemorrhagic fever. FEMS immunology and medical microbiology. 43(1), 99-102.

56. Jayashree K., Manasa G.C., Pallavi P., et al. 2011. Evaluation of Platelets as Predictive Parameters in Dengue Fever. Indian Journal of Hematology and Blood Transfusion. 27(3), 127-130.

57. Oliveira R., Cordeiro M.T., Moura P., et al. 2017. Serum cytokine/chemokine profiles in patients with dengue fever (DF) and dengue hemorrhagic fever (FHD) by using protein array. Journal of clinical virology. 89, 39-45.

58. Conroy A.L., Gelvez M., Hawkes M., et al. 2015. Host biomarkers are associated with progression to dengue haemorrhagic fever: a nested case-control study. International Journal of Infectious Diseases. 40, 45-53.

59. Lee M.S., Hwang K.P.,Chen T.C., et al. 2006.Clinical characteristics of dengue and dengue hemorrhagic fever in a medical center of southern Taiwan during the 2002 epidemic. J Microbiol Immunol Infect. 39(2), 121-9.

60. Khan M.I., Anwar E., Agha A., et al. 2013. Factors predicting severe dengue in patients with dengue Fever. Mediterranean journal of hematology and infectious diseases. 5(1), e2013014.

61. Saqib M.A.,Rafique I.,Bashir S., et al. 2014. A retrospective analysis of dengue fever case management and frequency of co-morbidities associated with deaths. [BMC Res Notes.](https://www.ncbi.nlm.nih.gov/pubmed/?term=A+retrospective+analysis+of+dengue+fever+case+management+and+frequency+of+co-morbidities+associated+with+deaths) 7, 205.

62. Soundravally R., Hoti S.L., Patil S.A., et al. 2014. Association between proinflammatory cytokines and lipid peroxidation in patients with severe dengue disease around defervescence. International journal of infectious diseases. 18, 68-72.

63. Weg C.A.M., Koraka P., Gorp E.C.M., et al. 2012. Lipopolysaccharide levels are elevated in dengue virus infected patients andcorrelatewith disease severity.Journal of Clinical Virology. 53, 38–42.

64. Humayoun M.A., Waseem T., Jawa A.A., et al. 2010. Multiple dengue serotypes and high frequency of dengue hemorrhagic fever at two tertiary care hospitals in Lahore during the 2008 dengue virus outbreak in Punjab, Pakistan. International journal of infectious diseases. 14(supp-S3), e54-e59.

65. Chau T.N., Anders K.L., Lien L.B., et al. 2010. Clinical and virological features of Dengue in Vietnamese infants. PLoS neglected tropical diseases. 4(4), e657.

66. Koraka P., Murgue B., Deparis X., et al. 2003. Elevated levels of total and dengue virus-specific immunoglobulin E in patients with varying disease severity. Journal of medical virology. 70(1), 91-8.

67. Wichmann O.,Hongsiriwon S.,Bowonwatanuwong C., et al. 2004. Risk factors and clinical features associated with severe dengue infection in adults and children during the 2001 epidemic in Chonburi Thailand. Trop Med Int Health.9(9), 1022-9.

68. Haryanto S., Hayati R.F., Yohan B., et al. 2016. The molecular and clinical features of dengue during outbreak in Jambi, Indonesia in 2015. Pathogens and global health. 110(3), 119-29.

69. Patra G., Ghosh M., Modak D., et al. 2015. Status of circulating immune complexes, IL8 titers and cryoglobulins in patients with dengue infection. Indian journal of experimental biology. 53(11), 719-25.

70. Mallhi T.H., Khan A.H., Adnan A.S., et al. 2015. Clinico-laboratory spectrum of dengue viral infection and risk factors associated with dengue hemorrhagic fever: a retrospective study. BMC infectious diseases. 15, 399.

71. Paranavitane S.A.,[Gomes L](https://www.ncbi.nlm.nih.gov/pubmed/?term=Gomes%20L%5BAuthor%5D&cauthor=true&cauthor_uid=25366086)., Kamaladasa A., et al. 2014. Dengue NS1 antigen as a marker of severe clinical disease. [BMC Infect Dis.](https://www.ncbi.nlm.nih.gov/pubmed/25366086)14, 570.

72. Senaratne T., Wimalaratne H., Alahakoon D.G., et al. 2016. Characterization of dengue virus infections in a sample of patients suggests unique clinical, immunological, and virological profiles that impact on the diagnosis of dengue and dengue hemorrhagic fever. Journal of medical virology. 88(10), 1703-10.

73. Vicente C.R., Herbinger K.H., Froschl G., et al. 2016. Serotype influences on dengue severity: a cross-sectional study on 485 confirmed dengue cases in Vitoria, Brazil. BMC infectious diseases. 16, 320.

1. Pone S.M., Hokerberg Y.H.M., de Oliveira R.V.C., et al. 2016. Clinical and laboratory signs associated to serious dengue disease in hospitalized children. J Pediatr (Rio J). 92(5), 464-71.
2. Wang WH, Lin CY, Chang K, et al. 2019. A clinical and epidemiological survey of the largest dengue outbreak in Southern Taiwan in 2015. Int J Infect Dis. Nov; 88:88-99.
3. Temprasertrudee S, Thanachartwet V, Desakorn V, et al. 2018. A Multicenter Study of Clinical Presentations and Predictive Factors for Severe Manifestation of Dengue in Adults. Jpn J Infect Dis. May 24;71(3):239-243.
4. Kuo HJ, Lee IK, Liu JW. 2018. Analyses of clinical and laboratory characteristics of dengue adults at their hospital presentations based on the World Health Organization clinical-phase framework: Emphasizing risk of severe dengue in the elderly. J Microbiol Immunol Infect. Dec;51(6):740-748.
5. Patra G, Mallik S, Saha B, Mukhopadhyay S. 2019. Assessment of chemokine and cytokine signatures in patients with dengue infection: A hospital-based study in Kolkata, India. Acta Trop. Feb; 190:73-79.
6. Agrawal VK, Prusty BSK, Reddy CS, et al. 2018. Clinical profile and predictors of Severe Dengue disease: A study from South India. Caspian J Intern Med. Fall;9(4):334-340.
7. Sehrawat P, Biswas A, Kumar P, et al. 2018. Role of Cytokines as Molecular Marker of Dengue Severity. Mediterr J Hematol Infect Dis. Apr 20;10(1): e2018023.
8. Patra G, Saha B, Mukhopadhyay S. 2019. Study of serum VEGF levels in patients with severe dengue infection admitted in a tertiary care hospital in Kolkata. J Med Virol. Oct;91(10):1873-1876.
9. Jayarajah U , Madarasinghe M , Hapugoda D, et al. 2020. Clinical and Biochemical Characteristics of Dengue Infections in Children From Sri Lanka. Global Pediatric Health, Nov 22;7:2333794X20974207.
10. Adil B, Rabbani A, Ahmed S, et al. 2020. Gall Bladder Wall Thickening in Dengue Fever - Aid in Labelling Dengue Hemorrhagic Fever and a Marker of Severity. Cureus. Nov 4;12(11): e11331.
11. Hegazi MA, Bakarman MA, Alahmadi TS, et al. 2020. Risk Factors and Predictors of Severe Dengue in Saudi Population in Jeddah, Western Saudi Arabia: A Retrospective Study. Am J Trop Med Hyg. Mar;102(3):613-621.
12. Shyamali NLA, Mahapatuna SD, Gomes L, et al. 2020. Risk Factors for Elevated Serum Lipopolysaccharide in Acute Dengue and Association with Clinical Disease Severity. Trop Med Infect Dis. Nov 16;5(4):170.
13. Maneerattanasak S, Suwanbamrung C. 2020. Impact of Nutritional Status on the Severity of Dengue Infection Among Pediatric Patients in Southern Thailand. Pediatr Infect Dis J. Dec;39(12): e410-e416.
14. Ayaz F, Furrukh M .2020. Assessment of Severity of Dengue Fever by Deranged Alanine Aminotransferase Levels. Cureus, Sep 19;12(9): e10539.
