# Supplementary material for: Risk and predictive factors for severe dengue infection: A systematic review and meta-analysis
Source: PLoS One. 2022 Apr 15;17(4):e0267186. doi: 10.1371/journal.pone.0267186 (PMC9012395; doi:10.1371/journal.pone.0267186)
Supplement: S4 Table — (DOC) [file pone.0267186.s005.doc]

**Table S4 Sensitivity and sub-analysis of co-variables on the summary effect and heterogeneity for factors with more than ten studies included**

| **Variable** | **Population** | **Data type** | **Diagnosis criteria** | **Study quality** | **Originated area** | **Sampling time** | **Sensitivity** |
| --- | --- | --- | --- | --- | --- | --- | --- |
| **Age** | Yes | No | No | No | No | No | NS |
|  | Homo | Hetero | Hetero | Hetero | Hetero | Homo |  |
| **Gender** | Yes | No | No | No | No | No | NS |
|  | Homo | Hetero | Hetero | Hetero | Hetero | Hetero |  |
| **DENV-1** | No | No | No | No | No | No | NS |
|  | Hetero | Hetero | Hetero | Hetero | Hetero | Hetero |  |
| **DENV-2** | No | No | No | No | No | No | NS |
|  | Hetero | Hetero | Hetero | Hetero | Hetero | Hetero |  |
| **DENV-3** | No | No | No | No | Yes | No | NS |
|  | Hetero | Hetero | Hetero | Hetero | Hetero | Hetero |  |
| **Day of illness** | No  Hetero | No  Hetero | No  Hetero | No  Hetero | No  Hetero | No  Hetero | NS |
| **Secondary infection** | No  Hetero | No  Hetero | No  Hetero | No  Hetero | No  Hetero | No  Hetero | NS |
| **Headache** | No | No | No | No | No | No | NS |
|  | Hetero | Hetero | Hetero | Hetero | Hetero | Hetero |  |
| **Osteodynia** | No | No | No | No | No | No | NS |
|  | Hetero | Hetero | Hetero | Hetero | Hetero | Hetero |  |
| **Retro-orbital pain** | No | No | No | No | No | No | NS |
|  | Hetero | Hetero | Hetero | Hetero | Hetero | Hetero |  |
| **Vomit** | No | No | No | No | No | No | NS |
|  | Hetero | Hetero | Hetero | Hetero | Hetero | Hetero |  |
| **Abdominal pain** | No | No | No | No | No | No | NS |
|  | Hetero | Hetero | Hetero | Hetero | Hetero | Hetero |  |
| **Hepatomegaly** | No | No | No | No | No | No | NS |
|  | Hetero | Hetero | Hetero | Hetero | Hetero | Homo |  |
| **Rash** | No | No | No | No | No | No | NS |
|  | Hetero | Hetero | Hetero | Hetero | Hetero | Hetero |  |
| **Petechiae** | No | No | No | No | No | No | NS |
|  | Hetero | Hetero | Hetero | Hetero | Hetero | Hetero |  |
| **Obvious bleeding** | No | No | No | No | No | No | NS |
|  | Hetero | Hetero | Hetero | Hetero | Hetero | Hetero |  |
| **Pleural effusion** | No | No | No | No | No | No | NS |
|  | Hetero | Hetero | Hetero | Hetero | Hetero | Homo |  |
| **Ascites** | No | No | No | No | No | No | NS |
|  | Hetero | Hetero | Hetero | Hetero | Hetero | Hetero |  |
| **Hypotension** | No | No | No | No | No | No | NS |
|  | Hetero | Hetero | Hetero | Hetero | Hetero | Hetero |  |
| **HCT** | No | No | No | No | No | No | NS |
|  | Hetero | Hetero | Hetero | Hetero | Hetero | Hetero |  |
| **PLT** | No | No | No | No | No | No | NS |
|  | Hetero | Hetero | Hetero | Hetero | Hetero | Hetero |  |
| **Low PLT*** | No | No | No | No | No | No | NS |
|  | Hetero | Hetero | Hetero | Hetero | Hetero | Hetero |  |
| **WBC** | No | No | No | No | No | No | NS |
|  | Hetero | Hetero | Hetero | Hetero | Hetero | Hetero |  |
| **HGB** | No | No | No | No | No | Yes | NS |
|  | Hetero | Hetero | Hetero | Hetero | Hetero | Hetero |  |
| **ALT** | No | No | No | No | No | No | NS |
|  | Hetero | Hetero | Hetero | Hetero | Hetero | Hetero |  |
| **AST** | No | No | No | No | No | No | NS |
|  | Hetero | Hetero | Hetero | Hetero | Hetero | Hetero |  |
| **CREA** | No | No | No | No | No | No | NS |
|  | Hetero | Hetero | Hetero | Hetero | Hetero | Hetero |  |
| **Alb** | No | No | No | No | No | No | NS |
|  | Hetero | Hetero | Hetero | Hetero | Hetero | Hetero |  |

Note:

No: no effect of co-variables on the result; Yes: there was a significant effect on the result by indicated co-variable

Homo: homogeneous result in the subgroup meta-analysis; Hetero: heterogeneous result in the subgroup meta-analysis

NS: the result was not significantly different when excluding any studies

* Dichotomous variables
