# Supplementary material for: Risk and predictive factors for severe dengue infection: A systematic review and meta-analysis
Source: PLoS One. 2022 Apr 15;17(4):e0267186. doi: 10.1371/journal.pone.0267186 (PMC9012395; doi:10.1371/journal.pone.0267186)
Supplement: S5 Table — (DOC) [file pone.0267186.s006.doc]

Table S5 Factors identified by subgroup for sampling time within seven days after onset of illness

| **Factors** | **Studies included** | **Sample size** | **Association with SD** | | **Test of Heterogeneity** | |
| --- | --- | --- | --- | --- | --- | --- |
|  |  | **(SD/DF)** | **OR/SMD (95% CI)** | ***p*-value** | ***I^2^*(%)** | ***p*-value** |
| secondary infection | 4 | 350/887 | OR =2.448  (0.955- 6.277) | 0.062 | 86.3 | <0.001 |
| hepatomegaly | 2 | 286/17642 | OR = 9.264  (7.034-12.201) | <0.001 | 0.0 | 0.402 |
| bleeding | 7 | 555/20461 | OR = 8.106  (3.094-21.241) | <0.001 | 92.4 | 0.000 |
| pleural effusion | 2 | 65/272 | OR = 87.143  (10.962-693.405) | <0.001 | 0.0 | 0.654 |
| ascites | 2 | 65/272 | OR = 83.578  (3.786-1844.938) | 0.005 | 49.3 | 0.160 |
| retro orbital pain | 3 | 108/304 | OR = 3.334  (1.228- 9.052) | 0.018 | 63.5 | 0.065 |
| AST | 7 | 526/19915 | SMD = 1.712  (0.276- 3.148) | 0.019 | 99.4 | <0.001 |
| platelet count | 10 | 667/20339 | SMD =-1.452  (-1.872- -1.031) | <0.001 | 94.1 | 0.001 |
| PLT* | 3 | 162/455 | OR = 48.931  (1.873- 1278.431) | 0.019 | 86.2 | 0.001 |
| HCT | 7 | 393/2620 | SMD = 0.706  (0.122- 1.291) | 0.018 | 95.2 | <0.001 |
| age | 5 | 334/720 | SMD =-0.048  (-0.192- 0.095) | 0.510 | 10.5 | 0.346 |
| gender | 9 | 720/1844 | OR = 1.081  (0.840- 1.393) | 0.545 | 41.0 | 0.094 |
| headache | 3 | 449/17922 | OR = 1.122  (0.667- 1.887) | 0.665 | 68.9 | 0.022 |
| Abdominal pain | 7 | 895/20504 | OR = 1.549  (0.960- 2.498) | 0.073 | 88.2 | <0.001 |
| diarrhea | 2 | 155/234 | OR = 0.623  (0.240- 1.615) | 0.105 | 31.2 | 0.228 |
| Vomiting | 6 | 434/2714 | OR = 1,917  (0.685- 5.365) | 0.215 | 93.6 | <0.001 |
| Osteodynia | 3 | 108/304 | OR = 2.145  (0.529- 8.704) | 0.285 | 84.5 | 0.002 |
| Myalgia | 4 | 226/571 | OR = 1.012  (0.500- 2.046) | 0.975 | 56.5 | 0.075 |
| rash | 5 | 341/962 | OR = 1.247  (0.747- 2.084) | 0.399 | 52.6 | 0.077 |
| Petechiae | 3 | 193/528 | OR = 3.611  (0.766- 17.022) | 0.105 | 89.4 | <0.001 |
| WBC | 7 | 523/18880 | SMD = 0.036  (-0.285- 0.357) | 0.827 | 88.2 | <0.001 |
| ALT | 7 | 430/18002 | SMD = 1.520  (-0.245- 3.284) | 0.091 | 99.4 | <0.001 |

* Dichotomous variables
