# Supplementary material for: Risk and predictive factors for severe dengue infection: A systematic review and meta-analysis
Source: PLoS One. 2022 Apr 15;17(4):e0267186. doi: 10.1371/journal.pone.0267186 (PMC9012395; doi:10.1371/journal.pone.0267186)
Supplement: S6 Table — (DOC) [file pone.0267186.s007.doc]

Table S6 Factors not associated with SD that were investigated in at least two studies

| Factors | NO. of study | Sample size  (SD/DF) | Model | Association with SD | | Test of Heterogeneity | | Publication bias  *p* value | |
| --- | --- | --- | --- | --- | --- | --- | --- | --- | --- |
|  |  |  |  | OR/SMD (95% CI) | *p*-value | *I^2^* (%) | *p*-value | Egger's | Begg's |
| Gender | 54 | 4856/30026 | Random | OR = 1.069(95% CI: 0.945-1.208) | 0.290 | 52.6 | <0.001 | 0.082 | 0.622 |
| SeroDENV-4 | 9 | 2307/3742 | Fixed | OR = 1.041(95% CI: 0.833-1.301) | 0.723 | 28.7 | 0.183 | - | - |
| Fever T | 7 | 337/2800 | Random | SMD = -0.113(95% CI: -0.452-0.226) | 0.515 | 84.6 | <0.001 | - | - |
| Headache | 24 | 2055/22289 | Random | OR = 0.847(95% CI: 0.590-1.216) | 0.367 | 83.4 | <0.001 | 0.540 | 1.000 |
| Weakness | 7 | 593/1488 | Random | OR = 1.839(95% CI: 0.762-4.436) | 0.175 | 48.3 | 0.071 | - | - |
| Osteodynia | 16 | 859/3477 | Random | OR = 1.113(95% CI: 0.773-1.602) | 0.565 | 60.4 | 0.001 | 0.588 | 0.837 |
| Myalgia | 20 | 1443/21039 | Random | OR = 0.930(95% CI: 0.749-1.155) | 0.512 | 35.9 | 0.056 | 0.238 | 0.289 |
| Retro-orbital pain | 16 | 1113/3502 | Random | OR =1.170(95% CI: 0.744-1.838) | 0.497 | 58.7 | 0.002 | 0.712 | 0.661 |
| Rash | 27 | 2236/25212 | Random | OR = 1.221(95% CI: 0.938-1.589) | 0.138 | 74.9 | <0.001 | 0.806 | 0.955 |
| Tourniquet test + | 6 | 469/968 | Random | OR = 1.857(95% CI: 0.923-3.736) | 0.083 | 75.2 | 0.001 | - | - |
| WBC | 23 | 1693/23759 | Random | SMD = 0.104(95% CI: -0.023-0.230) | 0.108 | 75.3 | <0.001 | 0.515 | 0.460 |
| Low WBC^#^ | 6 | 435/623 | Fixed | OR = 0.739(95% CI: 0.530-1.031) | 0.075 | 17.1 | 0.303 | - | - |
| HGB | 14 | 1212/21625 | Random | SMD = 0.109(95% CI: -0.009-0.227) | 0.070 | 54.7 | 0.007 | 0.064 | 0.228 |
| LYM | 5 | 452/2927 | Random | SMD = 0.029(95% CI: -0.312-0.371) | 0.866 | 87.1 | <0.001 | - | - |
| NEU | 5 | 314/1089 | Random | SMD = 0.051(95% CI: -0.706-0.809) | 0.894 | 95.0 | <0.001 | - | - |
| MON | 3 | 143/1025 | Random | SMD = 0.262(95% CI: -0.212-0.736) | 0.279 | 68.7 | 0.074 | - | - |
| ALP | 4 | 390/18225 | Random | SMD = 3.114(95% CI: -3.298-9.525) | 0.341 | 100.0 | <0.001 | - | - |
| High ALP^#^ | 2 | 131/202 | Fixed | OR = 1.489(95% CI: 0.406-5.459) | 0.548 | 32.9 | 0.222 | - | - |
| CREA | 12 | 888/21725 | Random | SMD = 2.562(95% CI: -1.680-6.830) | 0.237 | 100.0 | 0.025 | 0.827 | 0.347 |
| TBIL | 4 | 390/18225 | Random | SMD = 2.516(95% CI: -2.995-7.307) | 0.412 | 99.9 | <0.001 | - | - |
| Urine protein | 3 | 66/262 | Random | SMD = 0.804(95% CI: -0.401-2.010) | 0.191 | 80.1 | 0.025 | - | - |
| CHOL | 2 | 76/234 | Random | SMD = -0.839(95% CI: -1.998-0.319) | 0.155 | 94.1 | <0.001 | - | - |
| TG | 2 | 76/234 | Random | SMD = -0.677(95% CI: -2.920-1.566) | 0.554 | 98.4 | <0.001 | - | - |
| High PT^#^ | 2 | 139/206 | Fixed | OR = 0.854(95% CI: 0.470-1.550) | 0.603 | 0.0 | 0.367 | - | - |
| High APTT^#^ | 3 | 152/244 | Random | OR = 2.080(95% CI: 0.602-7.188) | 0.247 | 69.6 | 0.037 | - | - |
| D-dimer | 2 | 22/169 | Random | SMD = 1.521(95% CI: -0.282-3.324) | 0.098 | 92.4 | 0.000 | - | - |
| IFN-γ | 8 | 393/550 | Random | SMD = -0.031(95% CI: -0.333-0.271) | 0.840 | 72.2 | 0.003 | - | - |
| TNF-α | 6 | 330/446 | Random | SMD = 0.330(95% CI:0.025-0.635) | 0.034 | 69.9 | 0.005 | - | - |
| IFN-α | 2 | 86/69 | Random | SMD = -0.340(95% CI: -1.076-0.396) | 0.365 | 77.9 | 0.033 | - | - |
| IL-6 | 6 | 294/463 | Random | SMD = 0.288(95% CI: -0.042-0.617) | 0.087 | 72.8 | 0.002 | - | - |
| VEGF | 5 | 143/223 | Random | SMD =1.238(95% CI: -0.172-2.647) | 0.085 | 94.2 | <0.001 | - | - |
| IL-12P70 | 2 | 116/122 | Random | SMD = -0.546(95% CI: -1.695-0.604) | 0.352 | 92.5 | <0.001 | - | - |

Pooled odds ratios (OR) or standardized mean difference (SMD) with corresponding 95% confidence intervals (95%CI) of the published results were calculated.

#Factor was presented as a dichotomous variable.
