# Supplementary material for: Risk and predictive factors for severe dengue infection: A systematic review and meta-analysis
Source: PLoS One. 2022 Apr 15;17(4):e0267186. doi: 10.1371/journal.pone.0267186 (PMC9012395; doi:10.1371/journal.pone.0267186)
Supplement: Attachment — Submitted filename: Point-by-point response.docx [file pone.0267186.s009.docx]

Methods

Excluding non-English papers and abstracts may lead to biases – need to discuss

A: Yes, we added this limitation in the discussion section.

Lines 108-109 “Heterogeneity was considered statistically significant if the p-value≤0.10 and I^2^>50% [23,24].” It is unusual to choose a p value of 0.1, and why should the size of I-squared affect whether it is statistically significant? This needs further justification.

A: Yes, we agreed this point and amended it according to reference.

Were there any p-values which were>0.05 and ≤0.1? I could not see any. I suggest that p<0.05 is significant which is more usual practice.

A: There were two p-values (<0.05 and ≤0.1) used in our study and p≤0.1 is significant which is more usual practice in Cochran Q test according to the reference. (line106, line113, and line118)

Selection of fixed vs random effects based solely on I-squared is not appropriate. This should have been pre-specified based on expected clinical and methodological heterogeneity. (see Rucker et al, BMC Med Res Methodol. 2008; 8:79).

A: we agreed the point and a more practice way of the valuation of heterogeneity is often described as the Q-test and the *I^2^,* which was used in our study. (line 103-104)

Lines 112-113 – random effects model was used when I2>50% - not appropriate.

A: It was amended for >40% according to reference. (line 106)

Which outcomes were SMD used for? Blood cell count/hematacrit/lipids/prothrombin time/etc – were different units used? This needs to be explained as if not, then SMD is not the most appropriate method and mean difference would be preferable.

A: Continuous variables were analyzed by SMD and dichotomous data were analyzed by OR. The data of blood cell count, hematacrit, lipids, prothrombin time and etc has continuous and dichotomous variables and the units of these parameters were different.

Why were subgroups of Asia and America presented for each plot? Were these pre-specified? A rationale for splitting into geographic subgroups should be provided.

A: Yes, there were some published data showed that severe manifestations were different in the patients came from Asia and America. We added descriptions in the Methods section. (line 122-125)

What is table S3 showing? Is this factors associated with lowering severe dengue or factors not associated in any way? The table title “Negative factors associated with SDD that were investigated in at least two studies.” is confusing and unclear. I assume this should be “Factors not associated with sever dengue” table S3

A: Yes, these factors in table S4 were not associated with severe dengue. We changed the table title as *Factors not associated with SD that were investigated in at least two studies*, which would be feasible to understand the content in the table S4 for readers.

The sensitivity analyses are not well described. Table S4 is not explained and it is difficult to interpret. What are the definitions of homo and hetero in this table?

A: We added descript in Methods (line125-128) and Results section (line149-151) and added the definitions of homo and hetero in *Table S3*.

Results

More information needs to be given about serotypes. These are introduced in the results without any background to explain the relevance of this, and no descriptions on how this was done in the methods.

A: Yes, we agreed this point and added some descriptions in the discussion section. (line289-307)

Forest plots are poorly formatted, the labelling on the x-axis should be improved to indicate what positive and negative numbers suggest. The numbers on x-axis should be changed to something that does not look like a random number.

A: This was what the software showed when we conducted the outputs and we could not change the number on the x-axis.

Sensitivity analyses results are not described in the text。

A: We added descript in Results section. (line149-151)

Where are the results from the quality assessment? What was done with the studies of lowest quality or highest risk of bias? Were these excluded in a sensitivity analysis?

A: The results of quality assessment were showed in *Table S2*. The studies of lowest quality or highest risk of bias were excluded in a sensitivity analysis.

Other comments

DF and SDD not easy acronyms. Suggest to scrap acronyms and use dengue and severe dengue.

A: Amended.

English is often not easy to understand. For example, Lines 210-211 “However, publication bias in favour of positive studies was observed by Egger's test (p<0.05).” needs explaining. The manuscript needs proofreading generally.

A: The manuscript we are summitting now has been proofread by native English-speaking editors.

5. Review Comments to the Author

Reviewer #1: Please comments for improvement in the attached file.

The authors have demonstrated a well-executed methodology and statistical analysis. However, the methodology should be updated to only the latest classification. Another reason for the use of latest classification is to reduce the clinical heterogeneity of the data being analysed. Different classification might lead to different ways or focus of obtaining the clinical symptoms and signs by the clinician.

A: We do attend the classifications that would affect the heterogeneity of data extracted from different studies. However, manifestations of patients were obtained by clinicians personally and bias might be conducted even using the same classification. Therefore, we think it is more reasonable that all studies were included for analysis. We added descript in the introduction (line59-62) and discussion section (line272-275).

Reviewer #2: Kangzhuang Yuan and colleagues have investigated signs or biomarkers for early prediction of severe dengue. The authors summarized the main research question and key findings. The authors identified other literature on the topic and explained how the study relates to this previously published research. The manuscript is well organized and written clearly enough to be accessible to non-specialists. Overall, the study is well conducted and the manuscript is well written with the conclusions supported by the data. However, revision of the English language would be advisable. Thus, the manuscript shows sufficient potential for publication after some adjustments.

A: The manuscript we are summitting now has been proofread by native English-speaking editors.

Abstract

Line 20 - Typo: “severe dengue disease”

A: Amended. (line20)

Line 23 - The term "practical and inconsistent signs" is unclear and needs to be amended for clarity.

A: Amended. (line22-24)

Line 23 - Since SDD was already explained before, suggest using: “…for early prediction of SDD.”

A: Amended. (line23)

Line 25 - What is the period of the study? When did articles start to be selected? It is important to describe precisely the period in which the selection began and when it was finalized regarding each database. "

A: Amended. (line26)

Line 32 - Typo: “different”

A: Amended. (line33)

Line 33 - It is preferable to use "were not".

A: Amended. (line33)

Line 34 - Typo: “ascites”

A: Amended. (updated)

Line 34 - Typo: “associated”

A: Amended. (updated)

Line 38 - Suggest using “biomarkers”

A: Amended. (line35)

Line 38 - Perhaps more studies would be necessary for indicators to be considered established. Suggest to rewrite the phrase less emphatically.

A: We had rewritten the conclusions section. (line35-37)

Introduction

Line 44 - Suggest “living”

A: Amended. (line44)

Lines 46 and 47 - There is variance in the use of terms. Dengue refers to the clinical spectrum of dengue disease. Dengue fever, DHF, DSS, severe dengue, and dengue with warning signs are all labels created by various organizations to speak to disease severity based on clinical signs, symptoms, lab and other abnormalities. The authors should select a nomenclature and be consistent throughout.

A: Amended. (line42-43)

Line 50 - It would be recommended to update the data found in the literature.

A: We updated the data according the reference. (line46)

Lines 63 and 64 - Dengue disease is classified as dengue without warning signs, dengue with warning signs and severe dengue according to WHO guidelines (2009).

A: Amended. (line59-62)

Lines 68-69 - It is not clear what the authors are researching.

A: We amended the text. (line63-65)

Materials and Methods

Line 75 - Please inform the period in which the selection began and when it was finalized regarding each database.

A: Amended. (line71)

Line 95 - Table S1. It should be explained why the DHF/DW column for studies 1-12 is not completed. I suggest replacing the term “populations” (sixth column) with the term “age of patients”.

A: Amended. (line93)

Line 116 - Typo: “datasets”

A: Amended. (line112)

Line 117 - Typo: “did not”

A: Amended. (line113)

Results

Line 127 – In order to make it clearer to the reader, I suggest a list of the potential severity markers that have been studied and the signaling of those biomarkers that exhibited significant differences between DF and SDD.

A: Amended. (line133-137)

Line 134 - The study period should be described more accurately (month, year).

A: Amended. (140)

Line 143 - It should be explained what are the 62 factors to which the authors refer in the Methods section**.**

A: All 64 factors (two more factors, Day of illness and IFN-α were conducted after adding 13 studies) were presented in ***Table 1*** and positive factors were list in the first phase of results section (line133-137), therefore, we thought it was not necessary to list the details in this section.

Line 144 - Typo: “were not”

A: Amened. (line154)

Line 192 - Typo: “were not”

A: Amened. (updated)

Line 222 - Typo: “did not”

A: Amened. (line235)

Line 260 - It would be important to cite the studies in which the authors sought this information.

A: Amened. (line279-283)

Line 265 - There are studies showing that DENV-1 and DENV-3 were associated with severe dengue, with reported deaths from the disease in Brazil. Similarly, DENV-4 has not been associated with severe dengue in Brazil. It would be advisable to search the literature for further studies to support the findings.

A: We had amended the text according to more references. (line289-307)

Line 299 – Typo: “was not”

A: Amened. (line328)

Line 307 - Typo: “pathogeny”

A: Amened. (line336)

Line 308 - I suggest to indicate which literature studies the authors refer to.

A: We added references. (338)

Line 317 - It would be important to take into consideration some more factors such as: processing of samples into plasma or serum and WHO classification method used to assign the disease’s severity.

A：Yes, we agree this point but we can’t find any studies about the processing of samples. We added this suggestion as the study limitation in the discussion section. (line359-360)

Line 320 - Typo: “could not”

A: Amended. (line359-361)

Conclusion

Line 324 - It would be important for the authors to indicate which factors may be used as potential early laboratory biomarkers in the diagnosis of SDD.

A: Amended. (line363-369)

SDD might not be the commonly used abbreviation/term in recent published articles. Perhaps, ‘severe dengue (SD)’ alone is sufficient as it is a classification itself. If needed for a sentence, ‘severe dengue infection’ is preferred.

A: Amended.

The objective of the study is not well understood. Perhaps, the author should seek second opinion to revise the objective clearer, correcting the English language as well (grammar, verbs, etc). Overall the language used may be difficult to understand to the readers. I would suggest the manuscript to be proofread focusing on English and academic writing.

I would think the author should correct the language before I could go on reviewing the details of the manuscript. However, I would comment on the general aspect of the manuscript based on my assumption.

A: We rewrote the objective of this study and the manuscript was proofread for language by native English-speaking editors.

If I am correct, the author sought to explain the predictors by two components: clinical ‘symptoms/signs’ and laboratory ‘biomarkers’. Hence, the title may not truly represent the both as the title also includes epidemiological predictors such as weather, socioeconomic status, etc which could also predict the severity of the dengue. The title should be precise to reflect the objective of the study.

A: We amended the title.

I believe that the authors have missed relevant papers in their introduction/discussion like: “Zhang H, Zhou YP, Peng HJ, Zhang XH, Zhou FY, Liu ZH, Chen XG. Predictive symptoms and signs of severe dengue disease for patients with dengue fever: a meta-analysis. BioMed research international. 2014;2014.”

This paper is similar to one of the two components in the objective (clinical symptoms/signs). Thus, it is important to justify why the author’s study is needed since it has already been addressed and to compare and contrast in the Discussion section.

A: Zhang H, et al. conducted a meta-analysis that provided the evidence for the classifications of severe dengue disease according to the new WHO guideline 2009. They found that some symptoms and signs, bleeding, vomiting/nausea, abdominal pain, skin rashes, and hepatomegaly, maybe predictors for SDD. However, we do think that it is vital to investigate the predictors for SDD based on not only the symptoms but also biomarkers. Compared with symptoms, the plasma biomarkers results were obtained more objectively. We had added text in the Discussion section according to comparing Zhang’s study and ours. (line272-283)

The discussion section can have subheadings specifically to address the two components, thus addressing the objective clearly.

A: We added subheadings for each component in the discussion section. (line278, line288, line315, line353)

I recommend that the author should update the manuscript as 1 and half year has lapse since the last search. Many new articles that is relevant to the objective has been published. The work may be extensive but it is important that the systematic review should recommend the most current, up-to-date practice to the clinicians.

A: We had updated the literature searching period in this new manuscript.

I would suggest that articles which are not diagnosing with the latest WHO 2009 guideline should be excluded. Many countries have now adopted the new classification i.e. dengue without/with warning signs and severe dengue (SD). Any articles with DF, DHF, DSS can be excluded.

A: We do attend the classifications that would affect the heterogeneity of data extracted from different studies. However, manifestations of patients were obtained by clinicians personally and bias might be conducted even using the same classification. Therefore, we think it is more reasonable that all studies were included for analysis. We added descript in the introduction (line59-62) and discussion section (line272-275).
