# Supplementary material for: Risk and predictive factors for severe dengue infection: A systematic review and meta-analysis
Source: PLoS One. 2022 Apr 15;17(4):e0267186. doi: 10.1371/journal.pone.0267186 (PMC9012395; doi:10.1371/journal.pone.0267186)
Supplement: Attachment — Submitted filename: Response to Reviewers.docx [file pone.0267186.s010.docx]

Journal Requirements:

When submitting your revision, we need you to address these additional requirements.

1. Please ensure that your manuscript meets PLOS ONE's style requirements, including those for file naming. The PLOS ONE style templates can be found at
<https://journals.plos.org/plosone/s/file?id=wjVg/PLOSOne_formatting_sample_main_body.pdf> and
<https://journals.plos.org/plosone/s/file?id=ba62/PLOSOne_formatting_sample_title_authors_affiliations.pdf>

A: Yes, we amended in the main text.

2. Please revise your tables to replace p-values of "0.000" to "<0.001.

A：Amended in the main text and supplemental files.

3. Please attach a Supplemental file of the results of the quality assessment for each individual study assessed, reporting the outcome for each individual criteria considered.

A：The details of the quality assessment were shown in Table S2 which was uploaded as a supplemental file.

4. We note that the grant information you provided in the ‘Funding Information’ and ‘Financial Disclosure’ sections do not match.

When you resubmit, please ensure that you provide the correct grant numbers for the awards you received for your study in the ‘Funding Information’ section.

A: We would like to change the statement of financial disclosure and the details were included in resubmission cover letter.

5. In your Data Availability statement, you have not specified where the minimal data set underlying the results described in your manuscript can be found. PLOS defines a study's minimal data set as the underlying data used to reach the conclusions drawn in the manuscript and any additional data required to replicate the reported study findings in their entirety. All PLOS journals require that the minimal data set be made fully available. For more information about our data policy, please see <http://journals.plos.org/plosone/s/data-availability>.

Upon re-submitting your revised manuscript, please upload your study’s minimal underlying data set as either Supporting Information files or to a stable, public repository and include the relevant URLs, DOIs, or accession numbers within your revised cover letter. For a list of acceptable repositories, please see <http://journals.plos.org/plosone/s/data-availability#loc-recommended-repositories>. Any potentially identifying patient information must be fully anonymized.

Important: If there are ethical or legal restrictions to sharing your data publicly, please explain these restrictions in detail. Please see our guidelines for more information on what we consider unacceptable restrictions to publicly sharing data: <http://journals.plos.org/plosone/s/data-availability#loc-unacceptable-data-access-restrictions>. Note that it is not acceptable for the authors to be the sole named individuals responsible for ensuring data access.

We will update your Data Availability statement to reflect the information you provide in your cover letter.

A: All relevant data are within the manuscript and its Supporting Information files.

6. We note that you have stated that you will provide repository information for your data at acceptance. Should your manuscript be accepted for publication, we will hold it until you provide the relevant accession numbers or DOIs necessary to access your data. If you wish to make changes to your Data Availability statement, please describe these changes in your cover letter and we will update your Data Availability statement to reflect the information you provide.

A: We don’t have any data except that were within the manuscript and its Supporting Information files.

7. We note that you have included the phrase “data not shown” in your manuscript. Unfortunately, this does not meet our data sharing requirements. PLOS does not permit references to inaccessible data. We require that authors provide all relevant data within the paper, Supporting Information files, or in an acceptable, public repository. Please add a citation to support this phrase or upload the data that corresponds with these findings to a stable repository (such as Figshare or Dryad) and provide and URLs, DOIs, or accession numbers that may be used to access these data. Or, if the data are not a core part of the research being presented in your study, we ask that you remove the phrase that refers to these data.

A: Amended in the main text and provided relevant data.

8. Please upload a copy of Supporting Information Tables S1-S4 and Prisma checklist which you refer to in your text on page 20.

A: We had uploaded all supporting information Tables S1-S6, Prisma checklist, and raw data.

9. We note that this manuscript is a systematic review or meta-analysis; our author guidelines therefore require that you use PRISMA guidance to help improve reporting quality of this type of study. Please upload copies of the completed PRISMA checklist as Supporting Information with a file name “PRISMA checklist”.

A: Yes, we did.

10. Please review your reference list to ensure that it is complete and correct. If you have cited papers that have been retracted, please include the rationale for doing so in the manuscript text, or remove these references and replace them with relevant current references. Any changes to the reference list should be mentioned in the rebuttal letter that accompanies your revised manuscript. If you need to cite a retracted article, indicate the article’s retracted status in the References list and also include a citation and full reference for the retraction notice.

A: Yes, we checked all references again and all were correct.

[Note: HTML markup is below. Please do not edit.]

Reviewers' comments:

Reviewer's Responses to Questions

**Comments to the Author**

1. Is the manuscript technically sound, and do the data support the conclusions?

The manuscript must describe a technically sound piece of scientific research with data that supports the conclusions. Experiments must have been conducted rigorously, with appropriate controls, replication, and sample sizes. The conclusions must be drawn appropriately based on the data presented.

Reviewer #1: Yes

Reviewer #2: Yes

Reviewer #3: Yes

2. Has the statistical analysis been performed appropriately and rigorously?

Reviewer #1: Yes

Reviewer #2: Yes

Reviewer #3: N/A

3. Have the authors made all data underlying the findings in their manuscript fully available?

The [PLOS Data policy](http://www.plosone.org/static/policies.action#sharing) requires authors to make all data underlying the findings described in their manuscript fully available without restriction, with rare exception (please refer to the Data Availability Statement in the manuscript PDF file). The data should be provided as part of the manuscript or its supporting information, or deposited to a public repository. For example, in addition to summary statistics, the data points behind means, medians and variance measures should be available. If there are restrictions on publicly sharing data—e.g. participant privacy or use of data from a third party—those must be specified.

Reviewer #1: Yes

Reviewer #2: Yes

Reviewer #3: Yes

4. Is the manuscript presented in an intelligible fashion and written in standard English?

PLOS ONE does not copyedit accepted manuscripts, so the language in submitted articles must be clear, correct, and unambiguous. Any typographical or grammatical errors should be corrected at revision, so please note any specific errors here.

Reviewer #1: No

Reviewer #2: Yes

Reviewer #3: Yes

5. Review Comments to the Author

Please use the space provided to explain your answers to the questions above. You may also include additional comments for the author, including concerns about dual publication, research ethics, or publication ethics. (Please upload your review as an attachment if it exceeds 20,000 characters)

Reviewer #1: The manuscript “Risk and predictive factors for severe dengue infection: a systematic review and meta-analysis” by Yuan et al., offers a comprehensive review and reanalysis of the data from the manuscripts published on Dengue Fever, over the last 20 years. The focus of the manuscript is detection of the factors that are correlated with SD with the idea of using those factors to be able to better predict which patients with DF will progress to develop severe form of disease. Early detection and treatment of the severe form of Dengue Fever would lead to significant reduction of mortality.
Authors searched PubMed database for specific articles using very focused search words which yielded almost 7000 manuscripts. Through a multi-step filtering process, final number of 87 manuscripts were selected for inclusion in this study.
A very significant contribution that these types of analyses, and indeed this one as well, provide is closer examination of findings from individual studies in the context of much larger pool of data. Authors have used relatively stringent parameters to exclude a number of different correlates, but also provided stronger association for a number of other ones. Overall, as it would be expected, no new factors showing correlation with the SD were found, but it is possible that a significance of some factors has been emphasized.
Overall, as such, the manuscript does have a significant contribution to the field. However, there are few changes that can be made. By far, the most obvious one seems to be factoring the timing for the correlates. In the discussion, authors correctly state that it is critically important to identify the predictive factors for SD early, but nowhere in the manuscript they discuss timing for the correlates they mention. I am not sure what data is available on this, but even the crudest grouping would be helpful (early, late). Is there anything that can be concluded about the timing of warning signs relative to the onset of symptoms?
Besides that, there are few parts of the manuscript, most notably introduction and discussion where manuscript would benefit from more clarity in the way it was written. I think that the readability would be greatly enhanced if authors would consider having a technical writer or an editor review the grammar and the style.
With all this in mind, I would recommend publishing this, especially if the above changes are made.

A: We do agree that it is very important to identify the predictive factors for SD in the early stage of illness. Unfortunately, the sampling time in most studies were not clearly stated or just had an interval. Therefore, we performed a subgroup analysis for sampling time (we define “early stage” for ≤7 days after onset of illness) in the new manuscript we submitted. A total of 18 factors were identified and 9 revealed positive association with SD in the early stage of illness, which were added in the main text and shown in supplement file (Table S5).

The manuscript was reviewed by an English-speaker editor for grammar and style and all modifications were tracked in the main text.

Reviewer #2: I would like to thank and congratulate all authors for conducting timely important systematic review and meta analysis on “Risk and predictive factors for severe dengue infection: I believe results of this study will be much important mainly in resources limiting developing counties where their health resources are stretched to its maximum due to present pandemic.
I have few minor suggestions.
1. In the discussion section authors can mention novel bio marks such as Atypical lymphocyte count has been identified as a predictor for sever dengue infection.
2. Furthermore immature platelet fraction (IPF) too can be a predictor for SD.
3. Furthermore Triple positivity for nonstructural antigen 1, immunoglobulin M and immunoglobulin G is predictive of severe thrombocytopaenia related to dengue infection.
Above mentioned points can be included in discussion section . These may help on completeness of the study and planning of future reviews.

A: Yes, we got some studies focused on the three factors mentioned by reviewer. However, they could not be included the meta-analysis according to the including criteria of this study. Thus, we added some context in the discussion section referring to some references.

Reviewer #3: If the statistical reviewer is fine with the overall statistical conclusions, the article would be suitable for publication
The article would benefit from further english language editing and simplifying some of the difficult to understand sentences

A: The manuscript was reviewed again by an English-speaker editor for grammar and style and all modifications were tracked in the main text.

6. PLOS authors have the option to publish the peer review history of their article ([what does this mean?](https://journals.plos.org/plosone/s/editorial-and-peer-review-process#loc-peer-review-history)). If published, this will include your full peer review and any attached files.


If you choose “no”, your identity will remain anonymous but your review may still be made public.


**Do you want your identity to be public for this peer review?** For information about this choice, including consent withdrawal, please see our [Privacy Policy](https://www.plos.org/privacy-policy).

Reviewer #1: No

Reviewer #2: **Yes:** VisulaAbeysuriya

Reviewer #3: No

[NOTE: If reviewer comments were submitted as an attachment file, they will be attached to this email and accessible via the submission site. Please log into your account, locate the manuscript record, and check for the action link "View Attachments". If this link does not appear, there are no attachment files.]

While revising your submission, please upload your figure files to the Preflight Analysis and Conversion Engine (PACE) digital diagnostic tool, <https://pacev2.apexcovantage.com/>. PACE helps ensure that figures meet PLOS requirements. To use PACE, you must first register as a user. Registration is free. Then, login and navigate to the UPLOAD tab, where you will find detailed instructions on how to use the tool. If you encounter any issues or have any questions when using PACE, please email PLOS at [figures@plos.org](mailto:figures@plos.org). Please note that Supporting Information files do not need this step.
